# Single Dose Based Ertapenem Prophylaxis Reduces Surgical Site Infection after Selective Hepatectomy of Hepatocellular Carcinoma: A Propensity Score Matching Study

**DOI:** 10.1155/2018/2520191

**Published:** 2018-08-30

**Authors:** Bin Tang, Xiaolin Liu, Fei Xing, Chao Wang, Changjun Jia, Songlin Peng, Yang Zhao, Chaoliu Dai, Feng Xu

**Affiliations:** Department of Hepatobiliary and Splenic Surgery, Shengjing Hospital, China Medical University, China

## Abstract

**Objective:**

This study aimed to assess whether a single dose of ertapenem prophylaxis was more effective than other antibiotics to prevent surgical site infection (SSI) after selective hepatectomy for hepatocellular carcinoma (HCC).

**Methods:**

The data from HCC patients with open hepatectomy between January 2012 and June 2017 in Shengjing Hospital were retrospectively analyzed. These patients were divided into two groups: ertapenem (ER) group, where a single dose of ER was administered; non-ertapenem (NER) group, where NER antibiotics were administered. The SSI rates were compared between two groups before and after matching the propensity scores.

**Results:**

The enrolled patients consisted of 78 in the ER group and 197 in the NER group. After matching the propensity scores, each group was down-selected to 65 patients. The SSI rate among the matched 130 patients was 14.6%, 7.7% occurred in the ER group and 21.5% in the NER group (*P*<0.05). The SSI rates in organ/space of the ER and NER groups were 3.1% and 13.8%, respectively (*P*<0.05).

**Conclusions:**

A single dose of ER before surgery was more effective in mitigating SSI after selective hepatectomy compared with other antibiotics use. The results imply that the selection of both antibiotics and administration timing is important for the efficacy in preventing SSI.

## 1. Introduction

Hepatectomy remains as the curative treatment for liver cancer. Surgical site infection (SSI) is a common complication after liver resection. Although liver resection techniques, suture materials, and perioperative management have been greatly improved in recent years, SSI continues to occur in 3.1-14.0% of resected patients [1–8]. HCC in China predominantly occurs among patients with chronic liver disease and cirrhosis. Hepatic insufficiency, surgical bleeding, abnormal sugar tolerance, and weak immunity caused by liver resection are clearly risk factors for infection, and most of which are surgical site infection (SSI). SSI not only extends in-hospital stay, but also increases postoperative mortality [9, 10]. Many studies have been conducted to identify the risk factors associated with SSI [1, 8, 11–13] and devise effective prevention strategies [13–15].

It has been generally recommended in a clean or clean-contaminated procedure that antibiotics should be administered intravenously in the operative suite just before incision to prevent SSI [16]. Postoperative antibiotic administration has also been considered effective in preventing SSI after hepatectomy, which is a clean-contaminated surgery. Discontinuation of postoperative antibiotic administration within 24 hours postoperation is currently recommended. Many surgeons, however, tend to extend the duration of prophylactic antibiotic treatment. However, an extended prophylaxis with antibiotics does not necessarily improve the efficacy in prevention of infection. A prospective randomized controlled trial revealed that the two-day administration of flomoxef sodium was effective in reducing SSI after hepatectomy [15]. However, other randomized clinical trials revealed that postoperative antibiotic administration was not effective in preventing postoperative infections after liver resection, leading to an impression that antibiotic prophylaxis is not cost-effective and may not be deemed necessary [17, 18]. These differences may result from the faded function of different short-acting antibiotics, or/and low drug concentration in the plasma and tissues, which is not sufficient to inhibit bacterial infection. In theory, the administration of long-acting and effective concentrations of antibiotics should mitigate SSI after liver resection.

Another issue is that the over or frequent prescription of antibiotics may not provide the benefit of reducing SSI incidence at all. In contrast, it may cause bacterial resistance, leading to multiple infections.

Ertapenem is a member of carbapenem family with a broad-spectrum long-acting function that is generally indicated to treat infections, but not for prophylaxis. However, surgeons have recently started clinical trials with a single dose of ertapenem to prevent SSI in clean-contaminated surgery such as cholecystectomy and pancreatic resection in obese patients [19, 20]. The preliminary results revealed that a single dose of ertapenem can reduce SSI incidence. The concentration of ertapenem in liver tissues reached 5.28 mg/Kg in 240 minutes and remained up to 3.1 mg/Kg at 360 minutes after a single dose injection, which amounts to ≥90% of the pathogen minimum bacteriostatic concentration [21]. We hypothesized that ertapenem prophylaxis reduces SSI after hepatectomy, as it did in cholecystectomy. The present study aimed to evaluate the efficacy of a single dose of ertapenem within 30 minutes to two hours prior to skin incision in preventing SSI in patients with hepatocellular carcinoma (HCC).

## 2. Methods

### 2.1. Patients

Between January 2012 and June 2017, 396 HCC patients underwent open hepatectomy in Shengjing Hospital of China Medical University. Patients were excluded if they were treated with antibiotics within one week before surgery and had no perioperative antibiotic prophylaxis, emergency surgery, preoperative infection, extrahepatic metastasis, choledochojejunostomy, biliary tract exploration, digestive tract surgery, extrahepatic resection, or severe comorbidity such as pulmonary or renal insufficiency.

**Figure 1:**
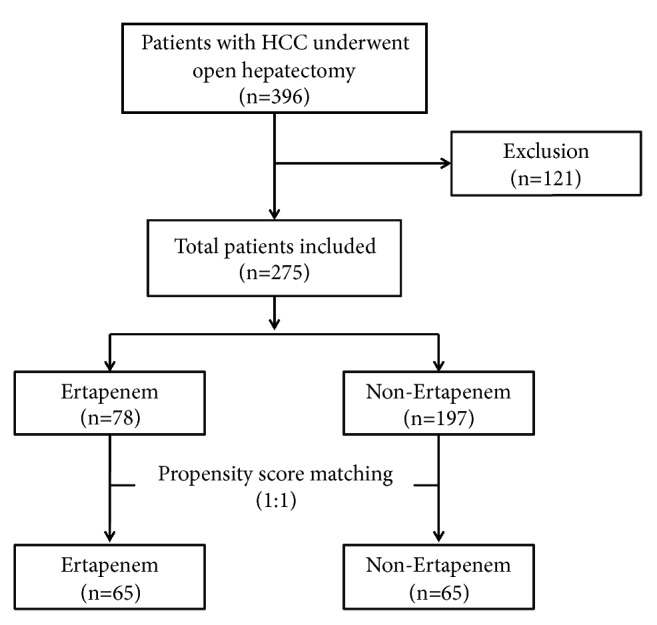
Flow chart of patient selection.

The exclusion resulted in the final enrollment of 275 patients. These patients were divided into two groups: ertapenem group (ER group,* n*=78) and non-ertapenem group (NER group,* n*=197) (Figure 1). Patients in the ER group were intravenously given a single dose (1.0 g) of ertapenem within 30 minutes to two hours prior to skin incision. Patients in the NER group were treated with a single dose of antibiotic prophylaxis of cefuroxime (1.5 g,* n*=43), cefoperazone (3.0 g,* n*=72), or piperacillin (4.5 g,* n*=82), preoperatively as in ER group, but antibiotic prophylaxis was used every 12 hours postoperatively until the temperature returned to normal.

### 2.2. Surgical Procedures

The resection methods and resection planes were selected based on tumor location, size, satellite nodules, presence or absence of macroscopic portal vein tumor thrombus, and liver function. Couinaud's segments were preferentially selected whenever possible when performing the anatomic resection due to its superior efficacy in oncological surgery [22]. Nonanatomic resection was featured with a negative tumor margin, regardless of segment or section anatomy. Major resection was defined as a resection that involved more than three Couinaud's segments. Liver parenchymal transection was performed using the clamp-crushing method or the use of an electrotome. The Pringle maneuver or hepatic blood inflow occlusion with hemihepatic artery retention was applied when necessary.

### 2.3. Postoperative Care

The drainage volume from the inserted abdominal tube was measured, and the fluid sample was submitted for laboratory testing and bacterial culture every two days after the operation. The abdominal drainage tube was kept* in situ* for 2–3 days and was gradually removed by withdrawing it at 1–2 cm length daily after confirming that the drainage fluid was aseptic. If patients developed fever, or/and abdominal symptoms, or had abnormal laboratory findings, ultrasound imaging was performed to scan the intra-abdominal space close to the raw surface of the liver remnant. CT imaging was acquired when an organ/space SSI was suspected. If the intra-abdominal fluid oozed or flowed, the patients were first treated with antibiotics. If antibiotic-based treatment was not possible or fails, a percutaneous drainage was performed under ultrasound guidance. When patients were discharged within one month after the resection, the monitoring of postoperative infection was conducted through visits to the clinic or telephone interview when it reached one month postsurgery.

### 2.4. SSI Diagnosis and Classification Criteria

SSI, which includes incisional infection and organ/space infection, was defined as an infection that occurred within 30 days postoperation, as advised by the National Nosocomial Infection Surveillance system [10]. Patients were classified as having incisional SSI if the infection involved the skin or subcutaneous tissue, or deeper soft tissues at the incision site. Deep organ/space infection was defined as the infection involved any part of the anatomy, other than the incision, that had been manipulated during surgery [23].

### 2.5. Propensity Score Matching (PSM) Analysis

In order to minimize patient selection bias and confounding variables between groups, a PSM analysis was conducted. All variables, except infection variables, were included in the matching model. A one-to-one nearest neighbor matching algorithm was applied with a caliper of 0.2 (PSM in SPSS®, version 1.0; F. Thoemmes, Cornell University, Ithaca, New York, USA).

### 2.6. Statistical Analysis

Statistical analyses were carried out using SPSS 22.0 for Windows (IBM, Armonk, New York, USA). Continuous variables were expressed as mean with standard deviation (SD). An independent* t*-test was used to compare continuous data. Categorical variables were presented as percentage, and *χ*^2^-test or Fisher's exact test was used for comparison. A two-tailed* P*<0.05 was considered statistically significant.

## 3. Results

### 3.1. Patient Characteristics

The baseline clinical data of patients in the ER and NER groups are shown in Tables 1 and 2. Before PSM, there were significant differences in prothrombin time, mean tumor size, and abdominal drain time between the two groups. In order to minimize patient selection bias and confounding variables between groups, a PSM analysis was conducted. After PSM (Supplementary Figure 1), differences in gender, age, BMI, smoking history, diabetes mellitus, preoperative TACE, secondary operation, albumin level, ALT level, Child-Pugh grade, total bilirubin level, operation method, gallbladder excision, operation time, intraoperative blood loss, blood transfusion amount, and bile leakage became insignificant between these two groups (*P*>0.05).

### 3.2. SSI Frequencies after Hepatectomy

The total infection and SSI rates were 28.36% (78/275) and 18.55% (51/275), respectively. The SSI rate was 9% and 22.3% in the ER and NER groups, respectively. This demonstrates the significantly more effective preventive efficacy of ertapenem (*P*=0.01). The effectiveness of ertapenem was further confirmed through PSM. After PSM, the SSI rate in the ER and NER groups was 7.7% (5/65) and 21.5% (14/65), respectively (*P*=0.025). Organ/space infection rate in the ER and NER groups was 3.1% (2/65) and 13.8% (9/65), respectively (*P*<0.05). The incisional infection rate was also lower (6.2%, 4/65) in the ER group, compared with the NER group (12.3%, 8/65). However, the* P*-value was >0.05 (Table 3). Among the 28 infected cases, 19 cases (ER group,* n*=5; NER group,* n*=14) were treated with antibiotics, and 3 organ/space infections (ER group,* n*=0; NER group,* n*=3) received the surgical intervention.

### 3.3. Isolation of Bacteria from Two Groups

Among the 28 infected cases, the bacteria were isolated in 20 cases by culturing vein blood, incision secretion, or abdominal drainage. The isolated bacteria included methicillin-resistant* Staphylococcus aureus* (MRSA),* Staphylococcus hominis*,* Staphylococcus epidermidis*,* Enterococcus faecium*,* Enterococcus hirae*,* Streptococcus constellatus*,* Klebsiella pneumoniae*,* Escherichia coli*,* Kluyvera ascorbata*,* Acinetobacter Bauman*,* Saccharomycopsis*, and* Candida tropicalis* (Table 4). The total rate of fungus or MRSA infections was significantly higher in the NER group comparing the ER group (*P*<0.05, Table 5).

## 4. Discussion

In the present study, we assessed the efficacy of the preoperative administration of ertapenem or non-ertapenem antibiotics in preventing SSI after open hepatectomy in HCC patients. These results revealed the SSI was significantly mitigated in the ER group, compared with the NER group, suggesting that ertapenem-based preoperative prophylaxis was more effective in preventing SSI than non-ertapenem antibiotics. This finding was further confirmed after the PSM between the ER and NER groups.

The higher efficacy in the ER group may have resulted from the high plasma concentration of ertapenem following an intravenous dose of 1 g. This is expected to maintain the plasma level at 1 mg/L, which is more than 90% of the minimum inhibitory concentration (MIC90), after 24 hours [24]. As reported, the same ertapenem dosage can be administrated, independent of gender, age, weight, or liver disease [24]. A prospective study assessed the efficacy of ertapenem in preventing infection after weight loss surgery and revealed that the incidence of postoperative infection did not increase among patients who received a single dose (1 g) of ertapenem injection before surgery, when compared with patients who received a preoperative injection of cefazolin (2 g) combined with intraoperative cefazolin (1 g) or with the preoperative administration of ampicillin (2 g)/sulbactam (1 g) [25]. As reported, the SSI rates in the ER group, ampicillin/sulbactam group, and continuous cefazolin group were 1.98%, 4.12%, and 1.50%, respectively. Another randomized controlled study assessed the efficacy of cefazolin sodium fluoride oxygen for infection prevention after liver resection and revealed that the administration of cefazolin sodium fluoride twice a day for three consecutive days after liver resection did not reduce the incidence of postoperative infection [17]. In the present study, the results revealed that the incidence of organ/space infection in the ER group was significantly lower than that in the NER group. Since the half-life of ertapenem in plasma is four hours, the ertapenem concentration in liver tissue at 360 minutes after a single dose injection was greater than 90% of the pathogen minimum bacteriostatic concentration [21]. The duration of effective drug concentration was sufficient to nearly cover the time typically required for the completion of the total hepatectomy in the present study.

The results of the present study also revealed that a fungus or MRSA infection was more likely to be present in the NER group after hepatectomy than in the ER group. Harbarth et al. also found that antibiotic resistance was 1.6 times higher in cases who were been treated with antibiotics for more than three days after surgery, compared to less than two days of treatment [26]. Therefore, excessive antibiotics (overdosed or prolonged course) likely promote drug resistance and multiple infections, including fungal and even possible MRSA infections. A randomized study revealed that there was no difference in overall infection, distant infection, and SSI incidence among patients with hepatic resection and 2- or 5-day treatment with flomoxef sodium [15]. The results from the latest randomized controlled study revealed that there was no difference in the incidences of postoperative overall infection, distant infection, and SSI between patient groups with 2- and 5-day postoperative antibiotics after major liver resection, combined with extrahepatic bile duct resection [27]. This suggests that the antibiotics regimen for preventing infection after hepatectomy should be as short as possible.

In the present study, the SSI rate in the NER group was higher than that of published data [1–6]. This may be related to the following factors. First, this study was a nonrandomized controlled study, and the sample size was relatively small, which may have skewed the frequency. Second, the prescribed antibiotics were divergent in the NER group, and the resultant efficacies may also be divergent in SSI prevention.

In general, the preoperatively preventive administration of antibiotics can reduce SSI after an operation. It is recommended to select antibiotics that are less likely to affect liver function and has a sufficiently long half-life that can cover the complete surgical procedure. There is great advantage for the use of ertapenem as an SSI prophylactic and as a single dose regimen, which minimizes the possibility of inducing drug resistance among the original bacteria or becoming susceptible to fungi or MRSA infection.

## Figures and Tables

**Table 1:** Comparisons of preoperative variables between the two groups before and after propensity score matching.

Variables	Before matching		*P* value	After matching		*P* value
	Ertapenem	Non-ertapenem		Ertapenem	Non-ertapenem	
	(*n*=78)	(*n*=197 )		(*n*= 65)	(*n*=65 )	
Age (year)	55.03 ± 10.24	55.84 ± 9.91	0.545	55.54 ± 10.11	54.11 ± 10.79	0.437
Gender (male/female)	60/18	159/38	0.482	49/16	54/11	0.28
BMI (kg/m^2^)	23.27 ± 3.09	22.98 ± 2.78	0.448	23.09 ± 3.15	23.16 ± 2.90	0.893
ASA grade, I/II/III	55/21/2	161/29/7	0.06	47/16/2	52/11/2	0.555
Virus hepatitis, positive/negative	72/6	176/21	0.456	59/6	57/8	0.571
Diabetes mellitus, yes/no	5/73	23/174	0.193	5/60	7/58	0.545
COPD, yes/no	4/74	22/175	0.123	4/61	2/63	0.403
Smoking history, yes/no	40/38	94/103	0.594	32/33	35/30	0.599
Preoperative TACE/RFA, yes/no	8/70	16/181	0.572	7/58	7/58	1
Preoperative intestinal lavage, yes/no	5/73	25/172	0.132	5/60	5/60	1
Repeat hepatectomy, yes/no	4/74	16/181	0.389	4/61	5/60	0.73
WBC(10^9^/L)	5.67 ± 2.05	5.67 ± 1.95	1	5.40 ± 2.01	5.72 ± 1.78	0.346
Hemoglobin (g/dL)	139.34 ± 21.90	140.45 ± 19.71	0.683	138.42 ± 23.06	141.35 ± 19.20	0.432
Platelet count (×10^4^/*μ*L)	16.12 ± 7.64	15.63 ± 7.11	0.614	15.72 ± 7.47	16.05 ± 7.77	0.805
Prothrombin time ( %)	12.52 ± 1.18	12.11 ± 1.20	0.011*∗*	12.46 ± 1.25	12.48 ± 1.26	0.917
Albumin (g/dL)	41.34 ± 4.63	40.72 ± 4.43	0.304	41.14 ± 4.79	41.26 ± 4.22	0.878
ALT (IU/L)	27.56 ± 11.72	30.36 ± 15.99	0.161	28.01 ± 12.19	29.58 ± 12.69	0.472
AST (IU/L)	34.81 ± 21.19	37.47 ± 43.25	0.605	36.28 ± 22.77	36.12 ± 31.14	0.974
Total bilirubin (mg/dL)	0.84 ± 0.44	0.87 ± 0.47	0.568	0.85 ± 0.47	0.77 ± 0.4431	0.262
Child-Pugh classification, A/B	71/7	173/24	0.448	58/7	62/3	0.188

**Table 2:** Comparisons of operative and postoperative variables between the two groups before and after propensity score matching.

Variables	Before matching		*P* value	After matching		*P* value
	Ertapenem	Non-ertapenem		Ertapenem	Non-ertapenem	
	(*n*=78)	(*n*=197 )		(*n*=65)	(*n*=65 )	
Tumor number, solitary/ multiple	71/7	163/34	0.082	59/6	55/10	0.286
Mean tumor size (mm)	40.32 ± 21.02	52.63 ± 45.80	0.023*∗*	41.66 ± 22.14	42.12 ± 21.08	0.903
Major hepatectomy, yes/no	18/60	41/156	0.68	16/49	17/48	0.84
Anatomical resection, yes/no	52/26	130/67	0.915	44/21	45/20	0.85
Cholecystectomy, yes/no	36/42	80/117	0.401	27/38	28/37	0.859
Mean operative time (min)	197.81 ± 71.69	195.02 ± 108.60	0.834	191.80 ± 66.68	191.72 ± 65.76	0.995
Mean blood loss (mL)	407.95 ± 348.58	657.87 ± 1293.94	0.094	421.54 ± 366.39	428.31 ± 513.44	0.931
Blood transfusion, yes/no	21/57	66/131	0.29	18/47	21/44	0.566
Hepatic inflow occlusion, yes/no	54/24	115/82	0.095	44/21	46/19	0.704
Postoperative hemorrhage, yes/no	1/77	4/193	0.675	1/64	0/65	0.315
Bile leakage, yes/no	5/73	12/185	0.921	5/60	7/58	0.545
Liver failure, yes/no	0/78	2/195	0.372	0/65	0/65	-
Ascites, yes/no	12/66	48/149	0.104	12/53	10/55	0.64
Pleural effusion, yes/no	11/67	34/163	0.524	10/55	11/54	0.812
Removal of abdominal drains (day)	9.13 ± 3.38	11.61 ± 8.36	0.012*∗*	9.32 ± 3.56	10.05 ± 5.40	0.369

**Table 3:** Comparison of infection between the two groups before and after propensity score matching.

Variables	Before matching		*P* value	After matching		*P* value
	Ertapenem	Non-ertapenem		Ertapenem	Non-ertapenem	
	(*n*=78) (%)	(*n*=197) (%)		(*n*= 65) (%)	(*n*=65 ) (%)	
Infectious complications	14 (17.9)	64 (32.5)	0.016*∗*	8 (12.3)	20 (30.8)	0.010*∗*
Remote site infections	9 (11.5)	30 (15.2)	0.429	5 (7.7)	7 (10.8)	0.545
Respiratory infection	7 (9.0)	20 (10.2)	0.767	3 (4.6)	5 (7.7)	0.465
Urinary tract infection	1 (1.3)	6 (3.0)	0.403	1 (1.5)	2 (3.1)	0.559
Catheter infection	3 (3.8)	9 (4.6)	0.792	1 (1.5)	2 (3.1)	0.559
Surgical site infections	7 (9.0)	44 (22.3)	0.010*∗*	5 (7.7)	14 (21.5)	0.025*∗*
Incision SSI	5 (6.4)	25 (12.7)	0.132	4 (6.2)	8 (12.3)	0.226
Organ/space SSI	4 (5.1)	25 (12.7)	0.066	2 (3.1)	9 (13.8)	0.027*∗*

**Table 4:** Isolated bacteria from two groups.

Variables	Ertapenem (*n*=8)	Non-ertapenem (*n*=20 )
Gram-positive cocci		
MRSA	0	2
*Staphylococcus hominis*	0	1
*Staphylococcus epidermidis*	1	0
*Enterococcus faecium*	0	1
*Enterococcus hirae*	0	2
*Streptococcus constellatus*	0	2
Gram-negative bacilli		
*Klebsiella pneumoniae*	2	5
*Escherichia coli*	1	3
*Kluyvera ascorbata*	0	1
*Acinetobacter Bauman*	0	1
Fungal infection		
*Saccharomycopsis*	0	1
*Candida tropicalis*	0	1
Negative	5	3

**Table 5:** Comparison of the total rates of MRSA or fungal infection between the two groups after propensity score matching.

Variables	Ertapenem (n=65)	Non-ertapenem (n=65)	*P* value
MRSA or fungal infection			0.042
No	65 (100)	61 (93.8)	
Yes	0 (0)	4 (6.2)	

## Data Availability

The data used to support the findings of this study are available from the corresponding author upon request.

## Supplementary Materials

Supplementary Materials
** Supplementary Figure **1: propensity score matching analysis.
