# Supplementary figures and images for: Single Dose Based Ertapenem Prophylaxis Reduces Surgical Site Infection after Selective Hepatectomy of Hepatocellular Carcinoma: A Propensity Score Matching Study
Source: Biomed Res Int. 2018 Aug 30;2018:2520191. doi: 10.1155/2018/2520191 (PMC6136539; doi:10.1155/2018/2520191)

## Supplementary Figure 1 Propensity score matching analysis

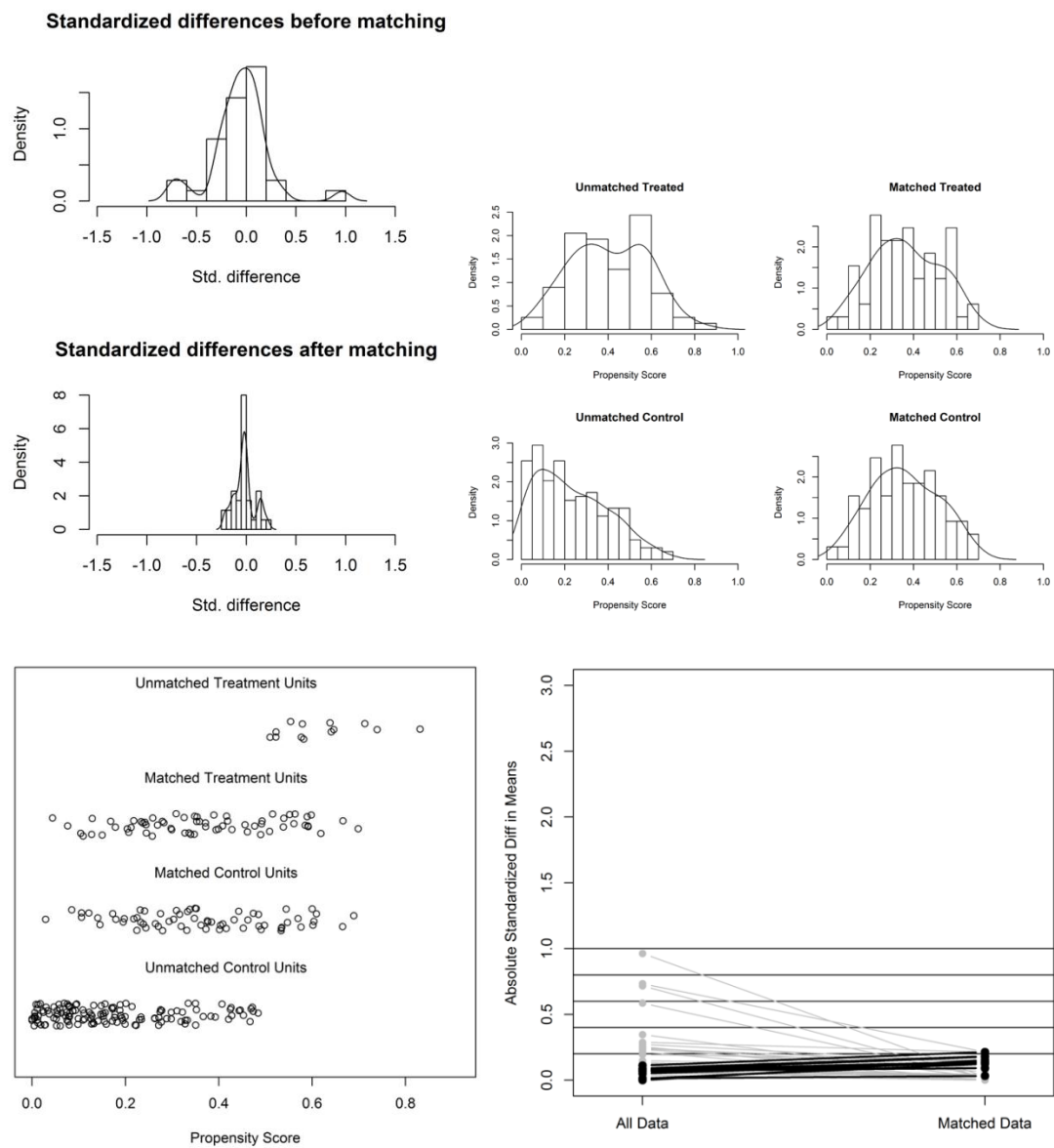

Supplement: Supplementary Materials — Supplementary Figure 1: propensity score matching analysis. [file 2520191.f1.pdf]
